# Comparative analysis of pediatric depression measures in clinical sample: evaluation of the PROMIS pediatric depression short form

**DOI:** 10.1007/s11136-025-04062-2

**Published:** 2025-10-11

**Authors:** Julia Rabin, Catherine Garcia-Goetting, Tareq Yaqub, Marina Teruel, John Lavigne, John Parkhurst

**Affiliations:** 1 Pritzker Department of Psychiatry and Behavioral Health, Ann & Robert H. Lurie Children’s Hospital of Chicago, Chicago, IL USA; 2 Department of Psychiatry and Behavioral Sciences, Northwestern University Feinberg School of Medicine, Chicago, IL USA

**Keywords:** Depression, PROMIS, Evidence-based assessment, Children

## Abstract

**Objective:**

Examine the psychometric properties of the PROMIS Depression Short From 2.0 (PROMIS-D-SF), Caregiver and Youth Report in comparison with a legacy measure of depression, the Short Mood and Feelings Questionnaire, using a clinical sample.

**Methods:**

Participants were 233 youth and caregivers referred to a behavioral health clinic by their pediatrician. Participants and their caregivers completed PROMIS-D-SF (youth and caregiver proxy), Short Mood and Feelings (SMFQ) (youth and caregiver proxy), and Semi-structured Interview. Descriptive, correlational, psychometric, receiver operating characteristic (ROC), and exploratory factor analysis were conducted for both measures.

**Results:**

PROMISD-SF youth and caregiver correlations with the youth and caregiver SMFQ were 0.73 and 0.68 and had Area Under the Curve (AUCs) values ranging from 0.7 to 0.77 for the detection of a depression diagnosis. The PROMIS-D-SF youth measure had favorable test re-test reliability and the factor structure of the PROMIS measure correlated with each factor of the legacy SMFQ measure.

**Implications:**

The PROMIS-D-SF demonstrated strong psychometric properties, correlated significantly with the SMFQ, and showed acceptable diagnostic accuracy for depression. This measure appears to be favorable for use in evidence-based assessment, including depression screening and progress monitoring.

**Supplementary Information:**

The online version contains supplementary material available at 10.1007/s11136-025-04062-2.

## Introduction

A major depressive episode occurs in 20.1% of adolescents (12–18) in the United States [1]. Depressive disorders (i.e. Major Depression, Dysthymia, Persistent Depression) occur in about 6.23% of adolescents and represent an increasing overall trend in prevalence from 2017 to 2021 in the United States [2]. Without treatment, depression can compromise the developmental process of youth and place them at increased risk for poor school performance, impaired social functioning, and lowered life satisfaction [3, 4].

Evidence-based assessment, including self and parent report measurement, is an essential first step in detecting symptoms of depression and providing appropriate treatment for pediatric depression [5]. After starting treatment, patient-report tools can promote progress monitoring to inform ongoing treatment decisions [6]. However, given that patient-report measures are typically underused in clinical practice due to a range of individual and systems level barriers (e.g., time, cost, lack of financial incentives from third party payers for screening) [7], additional research is necessary to aid clinicians in identifying measurement tools that are psychometrically sound, brief, and easy to implement.

The NIH developed PROMIS® (Patient-Reported Outcomes Measurement Information System) to provide rigorously tested, freely available self-report measures for diverse disorders [8]. Each measure was built using extensive item pools, refined for redundancy, translated, and psychometrically tested with item response theory [9]. Advantages of Item Response Theory, compared to other forms of mathematical modeling, include item invariance and greater detail on a measure’s precision [10]. For pediatric depression, the PROMIS Depression Short Form, Version 2.0 (PROMIS-D-SF) includes eight items for the pediatric patient and six items for their caregivers. While the rationale for the use of the PROMIS-D-SF in identification and progress monitoring of depression is strong, few studies have examined the factor structure and external validity through analysis with clinical populations [11]. Three recent studies [12–14] identified the PROMIS-D-SF as significantly associated with child mental health or psychopathology but did not compare the performance of the PROMIS-D-SF to other “legacy” measures of depression. In addition to limitations in our understanding of how well the PROMIS-D-SF compares to legacy measures, little is known about the sensitivity and specificity of clinical cut offs for identifying children who meet criteria for a depressive disorder [15].

The Short Mood and Feelings Questionnaire (SMFQ) is an established legacy measure of child and adolescent depressive symptoms [16, 17]. The SMFQ, designed for youth ages 8–18, has strong psychometric properties, is free to access and administer, includes parallel patient and caregiver-report forms, and has been widely used in clinical and epidemiological research [18–22]. This measure has established concurrent validity [23] and a unifactorial structure in community samples [16, 22–25]. Kuo et al., [26] reviewed the SFMQ’s factor structure within youth in juvenile detention, however, no other studies have identified the factor structure in a pediatric mental health sample.

The SMFQ has been used to determine whether children meet criteria for a diagnosis of depression. These studies have been conducted with community samples [20, 22, 27] and among children seeking help in mental health clinics [28, 29]. All but one study [28] was conducted with older children and adolescents only, ages 11 and older. These studies all showed Area Under the Curve values (AUC) indicating a least fair diagnostic accuracy (e.g., over 0.73, with most above 0.80), and with sensitivity (SE) of 0.70 or greater, and specificity (SP) of 0.68 or greater. Jarbin et al. [28] found poor AUCs, SEs, and SPs for child self-reports in the age 6–12 group, but somewhat better performance for parent reports of boys in this age range. Despite this base of literature for the SMFQ, cutoff scores varied substantially (see Supplemental Table 1).

### Study aims

The primary study aim was to assess the concurrent validity of the PROMIS-D-SF against the SMFQ, using an outpatient pediatric psychiatry sample. Along with concurrent validity, test–retest reliability of the PROMIS-D-SF was examined since we have been unable to identify studies describing this psychometric property in outpatient psychiatric populations. Secondarily, using a Receiver Operant Characteristic Analysis (ROC), we examine the diagnostic accuracy of the PROMIS-D-SF compared to that of the SMFQ in identifying a depressive disorder determined using an evidence-based semi-structured interview. Lastly, we assessed the structural validity and conceptual contextualization of the PROMIS-D-SF by exploring how the factor structure compares to that of the SMFQ in a clinical sample of children and adolescents seeking mental health care.

## Method

### Participants

Participants in this study were youth and their caregivers (*N* = 233) referred for a psychiatric evaluation by their primary care pediatricians. Participants for this study were referred by primary care pediatricians (between June of 2018 and October of 2024) participating in the Mood, Anxiety, and ADHD Collaborative Care (MAACC) Program, which was created to expedite mental health care access in primary care practices [30]. Youth (ages 5–18 years, *M* = 11.57, *SD* = 2.88) and their caregivers completed measures in English or Spanish.

### Measures

#### PROMIS pediatric depression short forms 2.0 (PROMIS-D-SF)

The PROMIS-D-SF measures were created to assess depression in youth ages 8–17 and their caregiver proxies for children ages 5–17. The measure uses a Likert scale of 1 through 5 (1 = never—5 = almost always) and total scores of the 8 items range from 8 to 40. Summed raw scores and their associated T scores (*M* = 50, *SD* = 10) in their most updated format can be found online on the health measures website. While no cut offs exist for PROMIS D-SF, existing pediatric research studies have described T score level of 62 as moderate to severe in pediatric samples of children with chronic illness and neurodevelopmental disorders [31–33].

### Short mood and feelings questionnaire (SMFQ)

The Short Mood and Feelings Questionnaire was developed to assess depression in the past two weeks with children 8–18 years of age [16, 17]. The SMFQ uses a Likert scale of 0 through 2 (0 = not true, 1 = sometimes, 2 = true) and total scores range from 0 to 26 for the 13-item measure, with higher scores indicating the presence of more severe depressive symptoms. Prior researchers have identified cut offs as low as 4 and as high as 12 to indicate the presence of depressive symptoms or a depression diagnosis [16, 20, 22, 26–29, 34]. The SMFQ also has strong criterion validity for children in early adolescence with a depressive disorder compared to structured interview [20].

#### Anxiety disorders interview schedule for DSM-IV: child and parent versions (ADIS-IV-C/P)

The ADIS-IV-C/P [35] is a semi-structured diagnostic interview for assessing psychopathology among youth ages 6–18. Clinical Severity Ratings (0 = not at all to 8 = very much) are assigned by the clinician for each diagnosis with the ADIS-IV-C/P, with a Clinical Severity Rating of 4 or greater representing symptoms and distress/interference at a level that meets full diagnostic criteria. The ADIS-IV-C/P is sensitive to clinical change and a valid and reliable measure [36] commonly utilized in clinical trials to assess children and adolescents for a range of DSM-IV disorders including depression.

### Procedure and data collection

Patients referred to MAACC were initially screened by telephone for appropriateness of referral. Patients were not eligible for MAACC if they were participating in higher level of care, diagnosed with Autism Spectrum Disorder Level II or III or global developmental delay. All patients and caregivers received the PROMIS-D-SF and SMFQ via paper or electronic system prior to the time of their diagnostic appointment. Patients whose measures were not available at the time of their appointment were asked to complete the PROMIS-D-SF and SMFQ via paper for clinical purposes. Data from this subset of patients, who later submitted the measures electronically was used for test re-test analyses. Based on the referral question and completed self-reported assessments, a licensed psychologist or post-doctoral fellow administered the relevant ADIS-IV-C/P modules with the child or adolescent and at least one caregiver/guardian. A Psychologist and Child Psychiatrist reviewed findings for all new diagnostic evaluations and collaborated on resulting diagnostic procedures outlined in Parkhurst et al. [30].

### Statistical analysis

All statistical analyses were conducted in SPSS version 28 [37], except for the Delong test, which was conducted in R [38, 39]. Independent T-Tests were used to assess any significant effect of demographic characteristics of race, ethnicity, and gender on PROMIS and SMFQ scores. Pearson correlations were conducted to assess covariance of SMFQ and PROMIS parent and youth measures. Correlation coefficients of < 0.39 or less are considered weak, 0.4–0.69 are considered moderate, and 0.7 and higher are considered strong [40]. To assess and compare the diagnostic ability of the PROMIS and SMFQ parent and youth measures, a Receiver Operating Characteristic (ROC) curve analysis was conducted and Area Under the Curve (AUC) values were extracted. AUC values of less than .5 indicate that the measure has no discriminatory power, 0.7–0.8 indicate fair or acceptable diagnostic accuracy, 0.8–0.9 indicate good to excellent diagnostic accuracy, and above 0.9 suggest outstanding diagnostic accuracy [41]. Although a sensitivity (SE) of >  = 0.85 and specificity (SP) of >  = 0.75 may be considered optimal, there is no well-established benchmark [42], and the choice of SE and SP depends on the way the tests and cutoffs might be used. At times, SE and SP are equally important and the choice of cutoffs will give equal weight to both. At other times, identifying cases may be more important even if SP will be lower. In the present study, we report SE and SP values when both are of equal importance and when an SE is considered more important with a value of 0.80 or higher.

A principal factors analysis (PCA) was conducted on PROMIS and SMFQ caregiver and youth measures to enable a direct structural comparison of the SMFQ with the PROMIS. This factor analysis was completed using Kaiser–Meyer–Olkin measurement (KMO) to confirm suitability of the factors, Principal Components Analysis for factor extraction, and Varimax rotation. Factors were extracted if they had an Eigenvalue greater than 1, and items with loading less than 0.5 were not included in the scales created for each factor.

## Results

### Descriptive statistics

As displayed in Table 1, approximately 70% of the sample identified as white (*n* = 157) and 13% as Hispanic (*n* = 30). The average age among participants was 11.57 (*SD* = 2.88). From the ADIS-IV semi-structured interview guide, 67 (29.30%) patients met criteria for depressive disorders, including Major Depressive Disorder, Major Depressive Episode, Mood Disorder Not Otherwise Specified, and Disruptive Mood Dysregulation Disorder. Of the total sample, 178 (77.70%) met criteria for at least one comorbid mental health condition. For youth, the mean score on the PROMIS D-SF was 19.12 (*SD* = 7.72) and the mean score on the SMFQ was 8.79 (*SD* = 6.23). The mean scores for caregivers were 13.68 (*SD* = 5.43) on the PROMIS D-SF and 7.29 (*SD* = 5.40) on the SMFQ.

**Table 1:** Sample characteristics

Characteristic	Overall (*n* = 233)
	*M (SD) or n (%)*
Age	11.57 (2.88)
Gender	
Male	107 (47.60%)
Female	115 (51.10%)
Other	3 (1.30%)
Ethnicity	
Hispanic	30 (13.40%)
Non-Hispanic	185 (82.60%)
Declined or Unknown	9 (4.00%)
Race	
White	157 (70.40%)
Black/African American	12 (5.40%)
Asian	6 (2.70%)
Biracial, Other	39 (17.50%)
Declined or Unknown	7 (3.10%)
American Indian	2 (0.90%)
Anxiety Diagnosis^a^	182 (79.50%)
OCD ^b^	12 (5.20%)
Depression Diagnosis ^c^	67 (29.30%)
ADHD Diagnosis ^d^	114 (49.80%)
ODD Diagnosis ^e^	15 (6.60%)
Trauma Diagnosis ^f^	11 (4.80%)
Co-morbid diagnosis (1 or more diagnosis)	178 (77.70%)
PROMIS D-SF Youth	19.12 (7.72)
PROMIS D-SF Caregiver	13.68 (5.43)
SMFQ Youth	8.79 (6.23)
SMFQ Caregiver	7.29 (5.40)

a = Includes the following primary, secondary, tertiary, and quaternary diagnoses: GAD, Separation Anxiety, Social Anxiety, or Panic Disorder; b = Includes the following primary, secondary, tertiary, and quaternary diagnoses: OCD; c = Includes the following primary, secondary, tertiary, and quaternary diagnoses: Depression (NOS), Major Depressive Disorder, Mood Disorder (NOS), Major Depressive Episode, Mood Disorder, Disruptive Mood Dysregulation Disorder; d = Includes the following primary, secondary, tertiary, and quaternary diagnoses: ADHD-Inattentive Type, ADHD-Hyperactive Type, ADHD-Combined Type; e = Includes the following primary, secondary, tertiary, and quaternary diagnoses: Disruptive Disorder, Conduct Disorder, Oppositional Defiant Disorder, Impulse Control Disorder; f = Includes the following primary, secondary, tertiary, and quaternary diagnoses: PTSD, Trauma, Adjustment Disorder, Bereavement, Other Trauma

Independent samples t-tests of youth and caregiver PROMIS-D-SF total score means and the youth and caregiver SFMQ total score means did not significantly differ by ethnicity. However, mean scores were significantly lower for white youth (*M* = 18.40, *SD* = 7.36) compared to non-white youth (*M* = 20.66, *SD* = 8.39) completing the PROMIS-D-SF (*t* = − 1.98; *p* = 0.05). Additionally, mean scores were significantly higher for non-male (e.g., female and non-binary identifying gender) (*M* = 9.73, *SD* = *6.61*) than male gender (*M* = *7.67, SD* = *5.42*) for the SMFQ youth (*t* = 2.26; *p* = 0.02).

Test re-test correlation coefficient as measured by Pearson’s correlation coefficient on the youth PROMIS-D-SF was *r* = 0.74 (*p* < 0.001, *n* = 57) and caregiver PROMIS-D-SF was *r* = 0.50 (*p* < 0.001, *n* = 48). The average length of time between tests was 0.88 weeks (6.16 days) with a range of 0–5.71 weeks.

### Concurrent validity

As displayed in Table 2, the youth PROMIS-D-SF correlated highly with the youth SMFQ (*r* = 0.733; *p* < 0.001). The caregiver PROMIS-D-SF had a moderately high correlation with the caregiver SMFQ (*r* = 0.681; *p* < 0.001). For caregiver child concordance, the PROMIS-D-SF youth had a moderate correlation with the PROMIS D-SF caregiver (*r* = 0.544; *p* < 0.001). For the SMFQ, the SMFQ youth had a moderately low correlation with the SMFQ caregiver (*r* = 0.442; *p* < 0.001).

**Table 2:** Correlations PROMIS and SMFQ for youth and caregiver forms

	Youth PROMIS depression SF	95% Confidence Interval (n = 217)	Caregiver SMFQ total	95% Confidence Interval (n = 211)
1. Youth SMFQ Total	0.733***	0.664–0.789	0.442***	0.327–0.545
		95% Confidence Interval(n = 227)		95% Confidence Interval (n = 224)
2. Caregiver PROMIS Depression SF	0.544***	0.446–0.630	0.681***	0.604–0.746

^*^p < 0.05; **p < 0.01; ***p < 0.001; Mean (SD)

### Diagnostic accuracy

AUC values of the PROMIS-D-SF and SMFQ (youth and caregiver versions) for diagnosing any depressive disorder are presented in Table 3. The AUC obtained for the Youth PROMIS-D-SF was fair for diagnosing any depressive disorder (0.77, 95% CI = 0.71–0.84). Similarly, the AUC obtained for the Caregiver PROMIS-D-SF was fair at 0.70 (95% CI = 0.62–0.77). For the Youth SMFQ, the AUC obtained was fair for diagnosing any depressive disorder (0.73, 95% CI = 0.66–0.80). The AUC for Parent SMFQ was poor at 0.64 (95% CI = 0.56–0.72). The difference between the AUCs of Parent SMFQ and Parent PROMIS-D-SF were statistically significant (*Z* = 2.33, *p* < 0.05). There was no significant difference between Youth SMFQ and Youth PROMIS-D-SF AUCs.

**Table 3:** Comparison of area under the curve and cutoff scores for the PROMIS and SMFQ

Scale	Diagnosis	*N*		AUC (95% CI)	T-Score	Cutoff	Sensitivity ((%)	Specificity (%)	Positive Likelihood Ratio
Youth PROMIS	Any Depression Disorder*	66	SE/SP balanced	0.77(0.71–0.84)	56.25	≥ 18.5	83.33	62.00	2.18
Youth PROMIS	Any Depression Disorder*	66	SE optimize	0.77(0.71–0.84)	55.10	≥ 17.5	86.40	55.10	1.91
Caregiver PROMIS	Any Depression Disorder *	67	SE/SP balanced	0.70 (0.62–0.77)	58.05	≥ 14.5	67.20	65.60	1.95
Caregiver PROMIS	Any Depression Disorder *	67	SE optimize	0.70 (0.62–0.77)	53.00	≥ 11.5	85.10	45.00	1.55
Youth SMFQ	Any Depression Disorder *	65	SE/SP balanced	0.73 (0.66–0.80)	–	≥ 7.50	69.20	60.70	1.76
Youth SMFQ	Any Depression Disorder *	65	SE optimize	0.73 (0.66–0.80)	–	≥ 4.5	90.80	38.70	1.47
Caregiver SMFQ	Any Depression Disorder *	64	SE/SP balanced	0.64 (0.56–0.72)	–	≥ 4.50	79.70	44.20	1.43
Caregiver SMFQ	Any Depression Disorder *	64	SE optimize	0.64 (0.56–0.72)	–	≥ 2.5	87.50	28.20	1.22

^*^Any Depression Disorder includes the following: Depression (NOS), Major Depressive Disorder, Mood Disorder (NOS), Major Depressive Episode, Mood Disorder, Disruptive Mood Dysregulation Disorder

Sensitivity, specificity, and positive likelihood ratios and associated clinical cut offs are displayed in Table 3 for the PROMIS-D-SF and SMFQ measures. These include cutoffs when SE and SP are considered equally important and cutoffs when SE is considered of greater importance (> = 0.85). When SE and SP were considered equally important, no PROMIS-D-SF or SMFQ measures generated a score meeting the desired 0.80 or greater sensitivity and specificity score of a screening measure; only the Youth PROMIS-D-SF approached the desired standard with an SE of 0.83 and SP of 0.62 (T-score of 56.25 and cutoff of 18.5). When SE was considered more important, an SE of >  = 0.85 could be identified for caregiver and child ratings of both PROMIS-D-SF and the SMFQ but SP values ranged from 0.28 to 0.55. Overall, the performance of the PROMIS-D-SF and SMFQ were similar for identifying children meeting criteria for any depressive disorder.

### Factor structure

A principal factor analysis (PCA) was performed for both the PROMIS-D-SF and the SMFQ using principal components analysis and orthogonal varimax rotation. Eigenvalues of ≥ 1 were used to interpret the number of factors in each measure and the minimum factor loading criteria was set to 0.50. The Kaiser–Meyer–Olkin (KMO) measure of sampling (0.88–0.93) and the Bartlett’s Test of Sphericity (*p* < 0.001) indicated the suitability of the PROMIS-D-SF and SMFQ youth and caregiver for factor analysis.

For the PROMIS-D-SF caregiver and youth measures, items loaded onto 1 factor that explained 71.8% and 66.37% of the total variance, respectively. For the SMFQ caregiver, 3 factors explained 62.38% of the total variance. For the SMFQ youth, 2 factors explained 54.31% of the total variance. Two items on the SMFQ youth and two items on the SMFQ caregiver failed to load significantly onto any factor so they were removed from the model. When re-running the SMFQ caregiver, findings confirmed the 3 factors, which explained 67.3% of the total variance. Our team identified these factors as Self-esteem (6 items), Anhedonia (3 items), and Concentration/Attention (2 items). When re-running the SMFQ youth without the two factors that did not load, findings confirmed two factors, which explained 58.7% of the variance. These factors were named Self-esteem (6 items) and Anhedonia (5 items). For all repeated exploratory factor analyses (EFAs), the KMO and Bartlett’s Test remained within suitable range to indicate that the data were suitable for factor analysis. Tables 4, 5, 6, 7, 8 and 9 show the total variance explained of the relevant factors for each measure and the factor loadings for the SMFQ caregiver and youth measures after rotation and removal of items. As displayed in Table 10, the one factor of the youth PROMIS-D-SF correlated moderately with the youth SMFQ self-esteem factor (*r* = 0.65; *p* < 0.001) and moderately with the SMFQ anhedonia factor (*r* = 0.63; *p* < 0.001). The one factor of the caregiver PROMIS-D-SF correlated moderately with the caregiver SMFQ self-esteem factor (*r* = 0.60; *p* < 0.001), moderately with the SMFQ anhedonia factor (*r* = 0.59; *p* < 0.001), and had a weak correlation with the SMFQ concentration/attention factor (*r* = 0.32; *p* < 0.001).

**Table 4:** Eigenvalues, percentages, and cumulative percentages of PROMIS-D-SF caregiver and youth

Measure	Factor	Eigenvalue	% of variance	Cumulative %
Caregiver PROMIS-D-SF	1	4.31	71.83	71.83
Youth PROMIS-D-SF	1	5.31	66.37	66.37

**Table 5:** Exploratory factor analysis of the PROMIS-D-SF caregiver and youth

Items	1
Caregiver PROMIS -D-SF	
1. My child felt everything in his/her life went wrong	0.86
2. My child felt lonely	0.89
3. My child felt sad	0.86
4. It was hard for my child to have fun	0.78
5. My child could not stop feeling sad	0.88
6. My child felt like he/she could not do anything right	0.82
Youth PROMIS D-SF	
1. I felt everything in my life went wrong	0.84
2. I felt lonely	0.84
3. I felt sad	0.79
4. It was hard for me to have fun	0.77
5. I could not stop feeling sad	0.79
6. I feel like I couldn’t do anything right	0.86
7. I felt alone	0.85
8. I felt unhappy	0.77

**Table 6:** Eigenvalues, percentages, and cumulative percentages of SMFQ caregiver

Factor	Eigenvalue	% of variance	Cumulative %
1	4.93	44.80	44.80
2	1.39	12.60	57.39
3	1.09	9.94	67.33

**Table 7:** Exploratory factor analysis of the SMFQ caregiver

Items	1	2	3
Factor 1: Self-esteem			
5. My child felt he/she was no good anymore	**0.79**		
8. My child hated him/herself	**0.77**		
9. My child felt he/she was a bad person	**0.82**		
11. My child thought nobody really loved him/her	**0.71**		
12. My child thought he/she could never be as good as other kids	**0.72**		
13. My child felt he/she did everything wrong	**0.73**		
Factor 2: Anhedonia			
1. My child felt miserable or unhappy		**0.71**	
2. My child didn’t enjoy anything at all		**0.78**	
3. My child felt so tired that he/she just sat around and did nothing		**0.83**	
Factor 3: Attention/ Concentration			
4. My child was very restless			**0.81**
7. My child found it hard to think properly or concentrate			**0.84**

The extraction method was principal axis factoring with varimax rotation. Factor loadings above 0.50 are in bold

**Table 8:** Eigenvalues, percentages, and cumulative percentages of SMFQ youth

Factor	Eigenvalue	% of variance	Cumulative %
1	5.35	48.63	48.63
2	1.11	10.06	58.68

**Table 9:** Exploratory factor analysis of the SMFQ youth

Items	1	2
Factor 1: Self-esteem		
8. I hated myself	**0.79**	
9. I was a bad person	**0.72**	
11. I thought nobody really loved me	**0.79**	
12. I thought I could never be as good as other kids	**0.73**	
13. I did everything wrong	**0.77**	
5. I felt I was no good anymore	**0.83**	
Factor 2: Anhedonia		
1. I felt miserable or unhappy		**0.61**
2. I didn’t enjoy anything at all		**0.68**
3. I felt so tired that I just sat around and did nothing		**0.73**
4. I was very restless		**0.62**
7. I found it hard to think properly or concentrate		**0.59**

The extraction method was principal axis factoring with varimax rotation. Factor loadings above 0.50 are in bold

**Table 10:** Correlations of the PROMI -D-SF with SMFQ factors

	SMFQ-Caregiver		
	SMFQ-Caregiver self-esteem factor (n = 224)	SMFQ Caregiver Anhedonia factor (n = 224)	SMFQ Caregiver Concentration/ attention factor (n = 224)
PROMIS-D-Caregiver (single factor)	0.60***	0.59***	0.32***

	SMFQ-Child		
	SMFQ-Child self-esteem factor (n = 217)	SMFQ-Child Anhedonia factor (n = 218)	
PROMIS-D-Child (single factor)	0.65***	0.63***	

^*^p < 0.05; **p < 0.01; ***p < 0.001

## Discussion

Given the prevalence of pediatric depressive episodes and disorders, identifying brief, valid measures that support identification and progress monitoring of depressive symptoms is foundational to evidence-based care. In the present study of youth referred to mental health evaluation from primary care, 29.30% (*n* = 67) of patients met criteria for depressive disorders on a semi-structured diagnostic interview (ADIS-IV), with 77.70% of the sample (*n* = 178) meeting criteria for one or more comorbid mental health condition.

The first aim of this study was to examine the concurrent validity of the PROMIS-D-SF by comparing it to the SMFQ. The youth and caregiver short form PROMIS-D-SF correlated well with respective youth and caregiver SMFQ measures, consistent with correlations between the PROMIS-D-SF and other legacy depression measures [32]. However, in alignment with prior literature on parent–child inter-rater reliability in depression assessment [43], correlations for parent child agreement of the PROMIS D-SF and SFMQ were weak. As such, prioritizing patient data in addition to caregiver data may be especially important in assessment contexts where agreement between patient and caregiver is less likely and caregivers are more likely to underreport internal emotional suffering [44].

We also sought to understand the extent to which the PROMIS-D-SF could detect depression, as compared to a gold standard measure (e.g. ADIS-IV). While optimal sensitivity and specificity was not achieved (SE = 0.8, SP = 0.8) by either PROMIS-D-SF measure, the youth PROMIS-D-SF measure demonstrated acceptable sensitivity (0.83) and specificity (0.62) using a T-score cut off of 56.25. These findings align with previous literature on the pooled values for sensitivity and specificity to rule out depression using brief screeners in adult cancer patients [32, 45]. However, the relative importance of optimizing sensitivity and specificity depends on the clinical setting and intended use of the screening tool. In some contexts, prioritizing a clinician's ability to correctly identify true positives and true negatives over minimizing false positives or false negatives may be more critical [46].

While semi-structured interviews like the ADIS-IV remain the gold standard for diagnosis, incorporated patient-reported outcomes can enhance integrated behavioral health settings by facilitating efficient, evidence-based assessment [47]. Evaluating the PROMIS-D-SF’s ability to predict depressive disorders in youth improves understanding of its diagnostic utility. By considering base rates alongside its sensitivity and specificity, clinicians can better assess the likelihood of depression beyond general prevalence estimates.

The third aim of the study was to examine the factor structure of the PROMIS-D-SF in relation to that of the SMFQ in a sample of children and adolescents referred for a mental health evaluation. In community samples, both measures appear to have a unidimensional factor structure [22, 48]. Factor structure, however, may vary with different samples, so our team proceeded with a principal factor analysis of both measures using our sample of children referred from pediatric primary care. The evaluation of the factor structure of both the PROMIS-D-SF and the SMFQ suggest additional areas of commonality and difference for these two measures. While the PROMIS-D-SF was unidimensional, the SMFQ had multiple factors. The three factors appeared to represent content that could be conceptualized as self-esteem, anhedonia, and concentration/attention. However, each of these SMFQ factors was significantly correlated with the single factor of the PROMIS-D-SF, providing additional evidence for the overlap in content of these two measures. Both measures incorporated content related to anhedonia, a cardinal feature of depressive symptomology [49].

Broadly, the PROMIS-D-SF appears to be a favorable measure for use in evidence-based assessment. The PROMIS-D-SF and SMFQ appear to measure similar depressive constructs. The PROMIS-D-SF's strong concurrent validity and acceptable diagnostic accuracy support this measure’s use in depression screening, particularly ruling out a depression diagnosis. Such findings suggest the utility of sequential screening for improved identification [50] particularly since positive predictive value may increase when measures are used in a two-step process [51]. The strong test–retest reliability of the PROMIS D-SF youth measure suggests that this measure is appropriate for use in progress monitoring and consistent over time.

### Strength and limitations

This study is the first to compare both the parent and youth PROMIS-D-SF to the parent and youth SMFQ and the gold standard diagnostic measure (ADIS-IV) in an outpatient pediatric psychiatry sample of over 200 patients. Additionally, our study is novel because of its provision of psychometric information on the PROMIS D-SF in a clinical population. While previous research has identified a unidimensional factor structure for the SMFQ, our study is the first to explore the SMFQ factor structure in a clinical sample. Findings support the use PROMIS-D-SF in clinical practice and suggest that the SMFQ’s depression factors, which correlate with PROMIS-D-SF, may differ between clinical and community samples.

This study has a few limitations. Inter-rater reliability among evaluators using the ADIS-IV was not assessed, and the SMFQ and PROMIS-D-SF measures were not blinded, introducing some potential bias in ADIS-IV module completion. Additionally, because the sample was predominantly non-Hispanic white, we could not conduct psychometric evaluations across different racial and ethnic groups. Since data for test–retest reliability was collected for the PROMIS-D-SF from both in-person and online survey administration, potential bias may have been introduced from this environmental and methodological difference across results. Thus, the test–retest correlation coefficients should be interpreted with caution.

## Conclusion

While the PROMIS-D-SF is a brief, psychometrically robust unidimensional screening tool, the SMFQ remains a widely used legacy measure for child and adolescent depression screening. The PROMIS-D-SF was significantly correlated with the SMFQ and the PROMIS-D-SF illustrated acceptable diagnostic accuracy for a depression diagnosis. Thus, the SMFQ and PROMIS-D-SF appear to be similar but not interchangeable measures. Given its efficiency and solid psychometric properties, the PROMIS-D-SF may be valuable for measurement-based care in high volume or resource limited clinical settings. Further research is needed to refine optimal cutoff scores, explore sequential screening strategies to enhance depression detection, and further explore factor structure differences for the PROMIS -D-SF across different psychiatric presentations in pediatric populations.

## Supplementary Information

Below is the link to the electronic supplementary material.

Supplementary Material 1

## Data Availability

The raw processed data required to reproduce the above findings can not be shared at this time as the data also forms part of an ongoing study.
