# Supplementary material for: Comparative analysis of pediatric depression measures in clinical sample: evaluation of the PROMIS pediatric depression short form
Source: Qual Life Res. 2025 Oct 11;34(12):3721–30. doi: 10.1007/s11136-025-04062-2 (PMC12689685; doi:10.1007/s11136-025-04062-2)
Supplement: Supplementary file 1 — Supplementary Material 1 [file 11136_2025_4062_MOESM1_ESM.docx]

**Supplemental Table**

*Current sensitivity and specificity of SMFQ from existing research*

| Author (Year) | Sample | *N* | Ages | Reporter | Gold Standard Diagnostic Measure | AUC | Sensitivity | Specificity | Cutoff |
| --- | --- | --- | --- | --- | --- | --- | --- | --- | --- |
| Kuo et al. (2005) | Youth in juvenile detention | 50 | 13-17 | Youth | DISC | 0.87 | 1 | 0.79 | >= 10 |
| Turner et al. (2014) | Community sample | 4027 | Mean = 17.9 | Youth | Clinical Interview Schedule-Revised | 0.87 | 0.712 | 0.83 | >= 11 |
| Katon et al. (2008) | Community sample | 1375 | 11-17 | Youth | DSIC | 0.84 | 0.8 | 0.81 | >= 6 |
| Thabrew et al. (2017) | Adolescents seeking treatment for depression | 183 | 11-21 | Youth | Child Depression Rating Scale-Revised, cutoff >= 44 | 0.86 | 0.842 | 0.682 | >= 12 |
| Rhew et al. (2010) | Community sample, 6th graders | 521 | 11-13 | Child  Parent  Parent + Youth | DISC | Child only, .73  Parent only, .74  Parent + Child, .86 | Child only, .66  Parent only, .66  Parent + Child, .76 | Child only, .61  Parent only, .66  Parent + Child, .78 | Child SMFQ, 4  Parent SMFQ, 4  Parent + Child SMFQ, 10 |

**Supplemental Table** *(continued)*

| Author (Year) | Sample | *N* | Ages | Reporter | Gold Standard Diagnostic Measure | AUC | Sensitivity | Specificity | Cutoff |
| --- | --- | --- | --- | --- | --- | --- | --- | --- | --- |
| Jarbin et al. (2020) | Mental health outpatients | 186 | 6-17 | Youth  Parent | Longitudinal expert all data (LEAD) diagnosis using K-SADS-PL 2009 | Girls (6-12), Child SMFQ = .51  Girls (6-12) Parent SMFQ = .73  Boys (6-12), Child SMFQ = .53  Boys (6-12), Parent SMFQ = .62  Girls (13-17), Child SMFQ = .82  Girls (13-17), Parent SMFQ = .85  Boys (13-17), Child SMFQ = .77  Boys (13-17), Parent SMFQ = .64 | Girls (6-12), Child SMFQ = .17  Girls (6-12) Parent SMFQ = .67  Boys (6-12), Child SMFQ = .29  Boys (6-12), Parent SMFQ = .63  Girls (13-17), Child SMFQ = .64  Girls (13-17), Parent SMFQ = .87  Boys (13-17), Child SMFQ = .93  Boys (13-17), Parent SMFQ = .76 | Girls (6-12), Child SMFQ = .96  Girls (6-12) Parent SMFQ = .83  Boys (6-12), Child SMFQ = .92  Boys (6-12), Parent SMFQ = .65  Girls (13-17), Child SMFQ = .96  Girls (13-17), Parent SMFQ = .76  Boys (13-17), Child SMFQ = .57  Boys (13-17), Parent SMFQ = .50 | Girls (6-12), Child SMFQ = 7  Girls (6-12) Parent SMFQ = 8  Boys (6-12), Child SMFQ = 14  Boys (6-12), Parent SMFQ = 7  Girls (13-17), Child SMFQ = 17  Girls (13-17), Parent SMFQ = 4  Boys (13-17), Child SMFQ = 6    Boys (13-17), Parent SMFQ = 6 |
